# Service Quality in Tourism Public Health: Trust, Satisfaction, and Loyalty

**DOI:** 10.3389/fpsyg.2021.731279

**Published:** 2021-08-30

**Authors:** Jiayu Han, Yifan Zuo, Rob Law, Sirong Chen, Mu Zhang

**Affiliations:** ^1^Shenzhen Tourism College, Jinan University, Shenzhen, China; ^2^Asia-Pacific Academy of Economics and Management, University of Macau, Macau, China; ^3^Department of Integrated Resort and Tourism Management, Faculty of Business Administration, University of Macau, Macau, China

**Keywords:** tourism public health, service quality, loyalty, trust, satisfaction

## Abstract

The spread of COVID-19 and large-scale travel restrictions has caused serious damage to the global tourism industry. Tourists pay additional attention to public health services and their health during travel, but studies on tourism public health service quality (TPHSQ) are limited. Therefore, this study aims to define TPHSQ and revise and validate its scale. The result of exploratory factor analysis (EFA) indicates that TPHSQ includes two dimensions “overall environmental image” and “public health facilities and management.” And based on 456 valid samples, the relationship among TPHSQ, tourists’ trust, satisfaction, and loyalty was validated using the multiple linear regression models. Results revealed the importance of the TPHSQ in improving tourists’ satisfaction and recovering their trust and loyalty. These results provided several implications for research, practice, and society that can benefit diverse stakeholders, which could accelerate the recovery of the tourism industry.

## Introduction

At the beginning of 2020, the COVID-19 epidemic has spread globally in just a matter of weeks. This unprecedented public health crisis, which is called a black swan event, has caused an adverse impact not only on public health but also on the tourism industry. According to the data released by the World Tourism Organization, the global tourism industry will lose $1.3trillion in revenue by 2020. In addition, the total number of global tourist arrivals will drop by 1billion, or 74%, compared with the previous year, making 2020 “the worst year in tourism history” (UNWTO, 2021). More and more tourists are unwilling or prohibited to travel because they are worried about being infected or threats to their personal safety during travel. Therefore, some scholars also predicted that after the outbreak, tourists will pay additional attention to public health services and their health and safety during travel (Wen and Jiang, 2020). In this context, tourism practitioners must clarify the importance of tourism public health services to reconnect with tourists and regain their confidence.

From the practical perspective, effective public health services and guidelines in the post-epidemic era play an important role in the recovery of the tourism industry. For example, in the United States, the NY State Park required visitors to practice social distancing by keeping at least six-feet distance from each other (The NY State Park, 2020). Moreover, in Japan, the Imperial Hotel installed thermal imaging cameras at the entrance to help measure guests’ temperatures and used trays to hold cash and credit cards during the payment process (Imperial Hotel Osaka, 2020). From the theoretical perspective, many previous studies showed that service quality can cause changes in many factors, such as satisfaction, trust, loyalty, and perceived risk (Melian-Alzola and Martin-Santana, 2020; Cong, 2021). Thus, in the tourism public health service context, this study predicted that a high-quality tourism public health service could improve tourist trust (TT) and satisfaction. Moreover, such a service could further influence travelers’ revisit intentions and recommendations, which is beneficial to help tourism destinations recover from this public health crisis.

However, most scholars only explored the importance of tourist destinations’ cleanliness and environment. Research related to tourism public health service quality (TPHSQ) and its impact on tourist loyalty (TL) is limited. For example, Zhou et al. (2015) found that destination cleanliness should obtain great attention from management. Hanafiah et al. (2019) also identified safety and cleanliness as important attributes of tourist experience quality. Using data on the rural TPHSQ, Sun (2018) verified that local tourism management departments should establish a feedback mechanism on the rural TPHSQ. Thus, the current study makes significant contributions to the literature by defining and measuring TPHSQ. The study also explores the mechanism of the influence of TPHSQ on tourists’ loyalty by using the multiple linear regression models and Bootstrap mediated effect test. The results will provide some managerial implications for destination management organizations (DMOs) from the perspective of tourism public health service in the post-pandemic era, even in the future.

## Literature Review

### Tourism Public Health Quality

Previous studies covered the aspects of the TPHSQ. Specifically, with regard to sanitation and hygiene factors, Milman (2009) evaluated the experience attributes of 608 tourists in the theme park. He confirmed that in addition to “service,” the most important factors are “courtesy, cleanliness, safety, and security.” He also showed that the “worshipping environment” is an extremely important part of the tourist experience. Then, for studies on public health facilities, Wang et al. (2020) explored the relationship between public environmental facilities and environmentally disturbing behaviors. They emphasized that tourism destination management departments should pay additional attention to public environmental facilities construction and immigration. For example, road construction should be reasonable, and the trash cans should be placed in places that tourists can easily find. Regarding the medical and health services during travel, Plentz (1978) proposed that protective vaccination in long-distance tourism is a problem of public health service. Previous studies always focused on one or some aspects of the TPHSQ but lacked a systematic definition and classification. Therefore, based on previous studies, this study obtained a clear definition of tourism public health service. That is, tourism public health service is a service provided by DMOs to improve tourist satisfaction (TS) and facilitate tourists to stay in a clean and healthy environment. Examples include providing a clean and hygienic tourist environment and necessary sanitation facilities and setting up a mature sanitation feedback mechanism. Meanwhile, the TPHSQ is the sum of the characteristics of this type of service.

In addition, although very few scholars studied this field, measuring TPHSQ is not very difficult because several proposals have been made on the multidimensional structure of service quality, such as the SERVQUAL scale (Parasuraman et al., 1988), SERVICESCAPE (Bitner, 1992), and SERVPERF (Cronin and Taylor, 1992). Among all these frameworks, SERVQUAL (Parasuraman et al., 1988) is the most successful assessment tool of service quality. This framework includes five dimensions, namely, assurance, reliability, tangibility, empathy, and responsiveness. Nonetheless, the dimensions of SERVQUAL were found to be not fully applicable to this research. Thus, this study referred to this scale and further revised it through exploratory factor analysis (EFA) to form a new TPHSQ Scale.

### Tourist Trust

Trust originated in psychology in the 1950s. Deutsch (1958) agreed that a trust is an act of believing in choice. In the context of tourism, tourist trust is widely accepted to play an important role in influencing their behavioral intentions. In other words, trust is somewhat predictive of tourist travel behaviors, such as revisit intention, commitment, and behavioral intention (Camara, 2019). In an earlier study, when people perceive a higher level of risk, trust has a stronger effect on creating behavioral intentions (Anderson and Karunamoorthy, 2003). Moreover, considering that public health service is closely related to each visitor’s health and safety, when the service involves their health, tourists tend to be more sensitive. Some relevant literature on medical tourism and health services also provided some evidence. For instance, Isa et al. (2019) proved that patients’ trust is having a direct relationship with perceived medical quality, relationship marketing, and word-of-mouth (WOM). Iranmanesh et al. (2018) stated that trust has a significant impact on the attitude of Muslim medical tourists to Islamic medical treatment.

### Tourist Satisfaction and Loyalty

Satisfaction and loyalty are not uncommon in tourism studies. In the 1970s, as the concept of “marketing” became more pervasive in the tourism industry, Pizam (1978) pioneered the concept of “tourist satisfaction,” which is the result of comparing tourists’ expectations before travel with their experience. Since then, many scholars have provided other concepts of satisfaction, mainly from the expectation theory and the emotional nature of experience. Similarly, the concept of tourist loyalty also comes from marketing and mainly consists of behavioral and attitudinal loyalty (Warshaw and Davis, 1985). Loyalty reflects the comprehensive evaluation of a destination’s products and services, including a tourist’s preference for the destination. Moreover, currently, in tourism studies, revisit intention and WOM recommendations are the most common indicators to measure loyalty.

### Hypothesis

Evidently, many scholars have verified the relationship between service quality and consumer loyalty, satisfaction, and trust from different areas, such as the in-flight services (An and Noh, 2009), mobile telecommunication industry (Aslam et al., 2018), and the most discussed by scholars, banking services (Lee and Moghavvemi, 2015; Fauzi and Suryani, 2019). In addition, in the unique field of blood donation, Melian-Alzola and Martin-Santana (2020) confirmed the impact of service quality on donor satisfaction and the effect of these two constructs on donor loyalty with a sample of 3,000 donors. This relationship is also not uncommon in the context of tourism. In the case of medical tourism, the significant positive relationship among the loyalty dimensions of revisit intention, recommendation, and positive recommendation has also been proven (Loke, 2020). Jeaheng et al. (2020) examined the relationship among halal-friendly hotel service quality and perceived price, satisfaction, trust, and revisit intention. Hence, although few scholars have explored the TPHSQ, we make the following hypotheses:

H1: TPHSQ has a positive and significant influence on tourist loyalty.

H2: TPHSQ has a positive and significant influence on tourist satisfaction.

H3: TPHSQ has a positive and significant influence on tourist trust.

Considerable academic debate about the relationship between trust and satisfaction exists. Although some studies (Al-Ansi and Han, 2019; Melian-Alzola and Martin-Santana, 2020) showed that customer satisfaction has a positive effect on trust, others showed that customers’ positive perception of trust affects their satisfaction (Chang, 2014; Wu et al., 2016; Amoako et al., 2019). In the field of tourism, Wu et al. (2016) found that trust is an important predictor of patient satisfaction in medical tourism. Amoako et al. (2019) also showed that customer satisfaction is influenced by trust and commitment in the hospitality industry.

Loyalty is one of the tourist intention behaviors. Many scholars verified that tourist trust can predict tourist intention behaviors (Wu et al., 2018). Therefore, the relationship between tourist trust and loyalty is also evident. Han and Hyun (2015) indicated that in a competitive medical tourism market; trust in the staff and clinic significantly affects tourists’ intentions to revisit clinics and the destination country. In addition, Jeaheng et al. (2020) found that trust has a positive and significant impact on Muslim guests’ revisit intention at the halal-friendly hotel. Hence, the following hypotheses were developed:

H4: Tourist trust has a positive and significant influence on tourist satisfaction.

H5: Tourist trust has a positive and significant influence on tourist loyalty.

Some pieces of empirical evidence support the relationship between satisfaction and loyalty in the tourism context (Priporas et al., 2017; Tanford and Jung, 2017). In addition, Bitner (1990) found that tourist satisfaction and loyalty are closely related. Therefore, destinations, tourism marketers, and local communities aim to generate satisfaction because of its strong relationship with loyalty (Oliver, 2014). If the tourists are less satisfied with the services, then they will not recommend or revisit the destination to a large extent. Thus, the following hypothesis is proposed:

H6: Tourist satisfaction has a positive and significant influence on tourist loyalty.

Previous studies found that tourists with higher satisfaction also have higher loyalty (Tanford and Jung, 2017). As mentioned earlier, several studies showed that tourism service quality and satisfaction are significantly related. Therefore, this study predicted that satisfaction will play a mediating role in the proposed theoretical model of the TPHSQ. This notion is similar to that of Peric et al. (2020), who reported that visitor satisfaction affects the relationship between service quality and loyalty. Moreover, a positive relationship is observed between service quality and tourist trust (Jeaheng et al., 2020). Additional research confirmed that as the trust increases, tourists are more likely to revisit the destination (Han and Hyun, 2015). Based on this, the following hypotheses are developed:

H7: Tourist satisfaction mediates the relationship between TPHSQ and tourist loyalty.

H8: Tourist trust mediates the relationship between TPHSQ and tourist loyalty.

**Figure 1:**
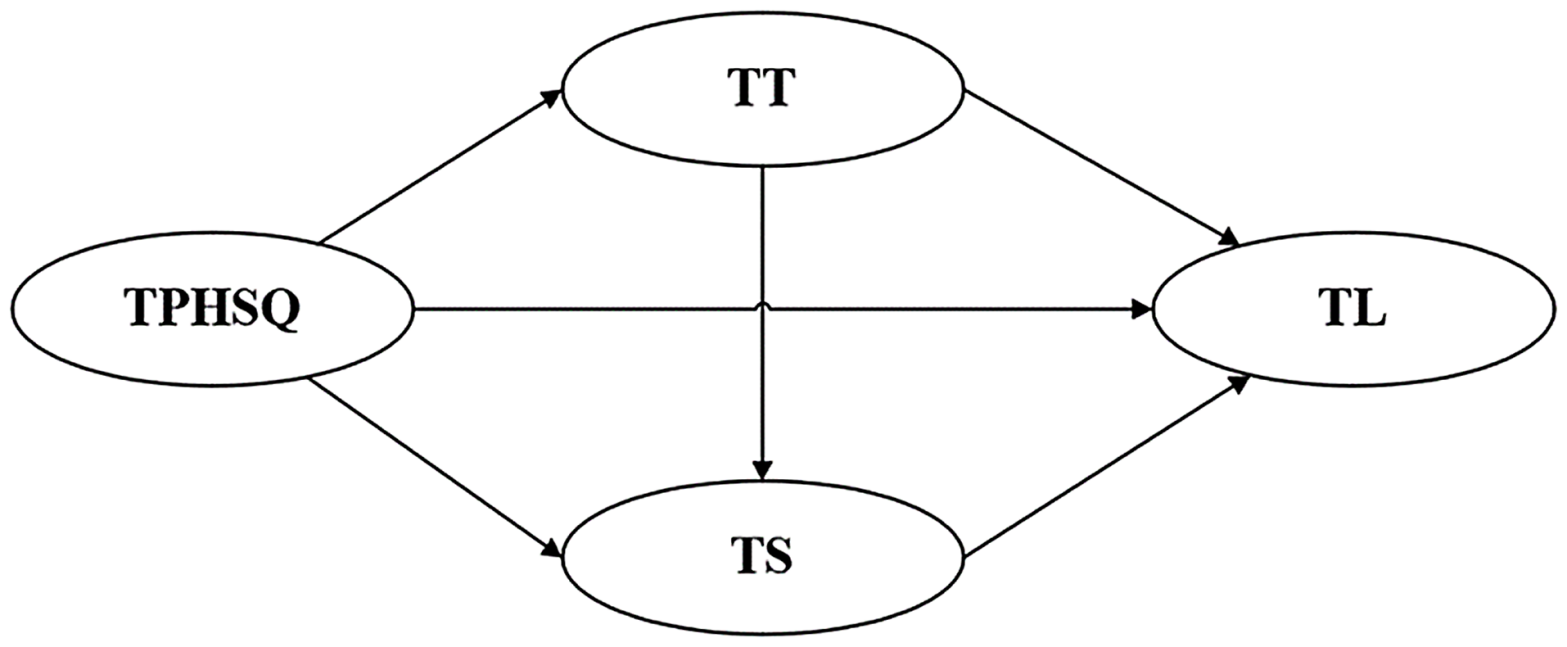
Proposed model. TPHSQ, tourism public health service quality; TT, tourist trust; TS, tourist satisfaction; and TL, tourist loyalty.

Figure 1 graphically illustrates the conceptual model in this study.

## Methodology

### Measures and Questionnaire Development

The questionnaire survey of this empirical study is divided into five parts. The first part mainly includes the demographic and personal background information of the participants. The second part is about the TPHSQ, mainly including the cleanliness of the environment and the public toilet, and whether to establish infirmaries and the public health service system in the scenic area. Then, the third part is used to measure the tourist trust, and the fourth and fifth parts are, respectively used to evaluate tourist satisfaction and loyalty after travel. Notably, we have set up two questions at the end of the first part of the questionnaire. One of which is “Which tourist attraction you have been to most recently?”, which could help tourists recall their most recent travel experience. Moreover, if they skip this question and do not answer, then the questionnaire will be considered invalid. The other question is a simple mathematical question, which is also used to test the validity of the data. All items were assessed using a five-point Likert-type scale ranging from extremely disagree (1) to extremely agree (5).

Based on the literature review, the measurement items for the study variables were adopted (Aydin and Özer, 2005; Yoon and Uysal, 2005; Lee et al., 2007; Paparoidamis and Chumpitaz Caceres, 2007; Bai and Guo, 2010; Žabkar et al., 2010; Guo et al., 2013). In particular, items regarding TPHSQ were adapted from previous studies (Sun, 2018). Moreover, the study was manually revised based on the original scale because of the limited universality and robustness of the original scale. First, 10 general visitors and two experts were invited to review the scale successively to check for accuracy. Second, we repeatedly discussed and compared their suggestions, and the original 38 measurement indicators were reduced to 16. Afterward, to ensure the scientificity and validity of the scale, the pilot test and EFA were conducted, and finally, a formal scale with 11 indicators was formed.

Previous research shaped other extended study variables, (e.g., tourist trust; Aydin and Özer, 2005; Paparoidamis and Chumpitaz Caceres, 2007; Guo et al., 2013) and included five items (e.g., “I trust completely the staff in this tourism destination.”). Then, tourist satisfaction toward this travel was measured with three scale items (e.g., “Overall, I am satisfied with my experience during this travel”), which were acquired from previous studies (Yoon and Uysal, 2005; Lee et al., 2007; Žabkar et al., 2010). Another three items were adapted for tourist loyalty (Bai and Guo, 2010), for example, “If I have the opportunity, I will promote the positive information of the destination to others.”

### Data Collection and Sample Characteristics

In this study, a convenience sampling method was used to distribute questionnaires through online social media platforms from December 17 to December 29, 2020. The link for participants to fill out online is https://www.wjx.cn/hj/4fv9udv8u0mxcf9ancaiza.aspx. Then, 593 questionnaires were eventually received, and of which, 456 were valid, having an effective rate of 76.9%. These samples came from 33 provinces, municipalities, and autonomous regions in China except Tibet. As shown in Table 1, 34.6% of the respondents (*n*=158) were male, and 65.4% (*n*=298) were female. Most of them were between 19 and 24years old, or 44.7% and those under 56years old accounted for 97.8%. In addition, more than half of the respondents, 62.9% (*n*=287), held an undergraduate degree. Table 1 summarizes the participants’ information.

**Table 1:** Sample profile.

Characteristics	*N*	%
**Gender**		
Male	158	34.6
Female	298	65.4
**Age**		
≤18	3	0.7
19–24	204	44.7
25–35	85	18.6
36–45	68	14.9
46–55	86	18.9
56–65	6	1.3
≥65	4	0.9
**Education level**		
Junior middle school and below	2	0.4
High school/Secondary school	30	6.6
Undergraduate	287	62.9
Master’s degree and above	137	30
**Occupation**		
Government officials	48	10.5
Company staff	52	11.4
Professional and technical personnel	61	13.4
Business personnel	2	0.4
Students	197	43.2
Mechanics/Workers	17	3.7
Waiters/Salesmen	4	0.9
Freelancers	25	5.5
Soldiers	13	2.9
Retirees	37	8.1
**Monthly income (RMB)**		
≤2,000	187	41
2,001–4,000	94	20.6
4,001–6,000	88	19.3
6,001–8,000	43	9.4
≥8,001	44	9.6

*N, number; %, percentage*.

### Data Analysis Tools and Procedures

At the beginning of this study, SPSS 22.0 was used to analyze the data and perform descriptive statistics on the demographic characteristics of the respondents. Next, the total sample (*N*=456) is divided into S1=150 and S2=306. The former is used for EFA and the reliability test, whereas the latter uses AMOS 22.0 software for confirmatory factor analysis (CFA) and hypothesis testing. Furthermore, to test the influence of multiple intermediaries on loyalty, this study used Hayes’s Model 6 and Bootstrap methods to conduct a series of intermediary analyses, verifying the intermediary role of tourist trust and satisfaction.

## Results

### Exploratory Factor Analysis and Reliability Test

This study employed EFA to measure TPHSQ and used principal components factor analysis and varimax rotation to perform factor extraction in SPSS software. Then, Cronbach’s alpha was used to check the internal consistency of each dimension. The results showed that the Cronbach’s alpha of each dimension of the TPHSQ initial scale was between 0.903 and 0.932, and that of each dimension exceeded the threshold of 0.75. This result indicates that the initial overall reliability of the scale and each dimension is very good, which is suitable for further analysis of other indicators.

Furthermore, values from the Kaiser–Meyer–Olkin measure of sampling adequacy and Bartlett’s test of sphericity were obtained to verify the appropriateness of factor analysis in the data. The Kaiser–Meyer–Olkin measure of 0.918 indicates that the factor analysis is suitable based on the 0.8 guideline, which interprets 0.8 and above as meritorious (Hair et al., 2010). From another perspective, the Bartlett test of sphericity of 0.00 also indicates that the variables are suitable for factor analysis.

Table 2 shows that the two factors are named “Overall environmental image” and “Public health facilities and management,” with a cumulative variance contribution of 72.096%. In the EFA, after the maximum variance is orthogonally rotated, the item with the factor loading value of less than 0.4 and the loading value on two or more factors simultaneously is greater than 0.4 should be deleted (Maskey et al., 2018). According to the above standards, after several rounds of deletion, the scale finally retained 11 items and deleted five.

**Table 2:** Exploratory factor analysis (EFA) and reliability test.

	Item	Factor loading	Mean	Weight (%)	Eigenvalues	Variance contribution rate (%)	*α*
Overall environmental image	TPHSQ1	0.756	3.94	8.714	1.364	8.527	0.903
	TPHSQ2	0.802	3.67	9.244			
	TPHSQ3	0.841	3.65	9.693			
	TPHSQ4	0.859	3.54	9.901			
Public health facilities and management	TPHSQ5	0.822	3.72	9.474	10.171	63.569	0.932
	TPHSQ6	0.787	3.58	9.071			
	TPHSQ7	0.83	3.35	9.567			
	TPHSQ8	0.661	2.92	7.619			
	TPHSQ9	0.793	3.10	9.140			
	TPHSQ10	0.744	3.22	8.575			
	TPHSQ11	0.781	3.30	9.002			

*The items can be found in*Appendix A.

### Confirmatory Factor Analysis

Confirmatory factor analysis was performed by using AMOS software. As shown in Table 3, the Cronbach’s alpha value is between 0.800 and 0.893, and all constructs exceed the threshold of 0.75, indicating that the internal consistency among each scale is acceptable (Christmann and Van Aelst, 2006).

**Table 3:** Confirmatory factor analysis (CFA) and reliability analysis.

Variables and items	M	SD	CITC	Cronbach’s α	Factor loading	AVE	CR
**TPHSQ**							
TPHSQ1	4.072	0.798	0.535	0.893	0.574	0.411	0.884
TPHSQ2	3.725	0.925	0.586		0.605		
TPHSQ3	3.856	0.864	0.590		0.632		
TPHSQ4	3.755	0.807	0.601		0.61		
TPHSQ5	3.788	0.922	0.580		0.634		
TPHSQ6	3.696	0.928	0.714		0.7		
TPHSQ7	3.428	0.960	0.682		0.672		
TPHSQ8	2.961	1.033	0.497		0.539		
TPHSQ9	3.15	0.939	0.675		0.696		
TPHSQ10	3.324	0.918	0.685		0.692		
TPHSQ11	3.353	0.961	0.664		0.675		
**TT**							
TT1	3.621	0.781	0.711	0.875	0.757	0.577	0.872
TT2	3.451	0.801	0.688		0.769		
TT3	3.552	0.784	0.742		0.75		
TT4	3.487	0.765	0.695		0.779		
TT5	3.513	0.790	0.684		0.742		
**TS**							
TS1	3.618	0.798	0.652	0.800	0.767	0.582	0.807
TS2	3.493	0.803	0.659		0.761		
TS3	3.539	0.764	0.626		0.761		
**TL**							
TL1	3.425	0.866	0.603	0.800	0.752	0.580	0.806
TL2	3.562	0.754	0.680		0.764		
TL3	3.641	0.811	0.660		0.769		

*The variables and items can be found in*Appendix A. *M, mean; SD, standard deviation; CITC, corrected item-total correlation; AVE, average variance extracted; CR, composite reliability; TPHSQ, tourism public health service quality; TT, tourist trust; TS, tourist satisfaction; and TL, tourist loyalty*.

The results also showed that the final CFA model has good fitting index (χ^2^=221.17, df=170, χ^2^/df=1.301, RMSEA=0.031, GFI=0.940, NFI=0.963, IFI=0.991, TLI=0.988, and CFI=0.991). The CR values for all the constructs ranged from 0.806 to 0.884, which exceeded the threshold value of 0.70 (Hair et al., 2010), and the factor loading of each item is between 0.5 and 0.9. Moreover, as the average variance extracted (AVE) values from all constructs ranged from 0.411 to 0.582, most of them are greater than 0.5 (Fornell and Larcker, 1981). This result shows that the scale has good convergent validity. Then, as shown in Table 4, the square root value of AVE is greater than the correlation coefficient of each and other variables, indicating that each dimension has high discriminative validity.

**Table 4:** Correlation coefficient matrix.

Variable	TPHSQ	TT	TS	TL
TPHSQ	0.641			
TT	0.297^***^	0.760		
TS	0.300^***^	0.377^***^	0.763	
TL	0.345^***^	0.433^***^	0.448^***^	0.762

*Diagonal elements show the square root of AVE. Below the diagonal is the corresponding correlation coefficient. All correlation coefficients were significant at the 0.01 level. TPHSQ, tourism public health service quality; TT, tourist trust; TS, tourist satisfaction; and TL, tourist loyalty*.

*** *Significant at <0.01*.

### Hypothesis Testing

This study predicted that improving the TPHSQ would increase tourist loyalty (H1), trust (H2), and satisfaction (H3). The results showed that TPHSQ had a significant positive effect on loyalty (*B*=0.172, *p*<0.05), and TPHSQ significantly and positively affected tourist trust (*B*=0.972, *p*<0.05) and satisfaction (B=0.341, *p*<0.05) as shown in Table 5.

**Table 5:** Regressions analysis.

DV	IVs	*B*	S.E.	*t*	*p*	95% CI		Hypothesis
						LLCI	ULCI	
TT	TPHSQ	0.972	0.015	64.804	0.000	0.942	1.001	H3
	R^2^	0.933	*F* = 4199.5068, *p* <0.0001					
TS	TT	0.677	0.057	11.796	0.000	0.564	0.789	H4
	TPHSQ	0.341	0.058	5.906	0.000	0.227	0.454	H2
	R^2^	0.938	*F* = 2285.4645, *p* <0.0001					
TL	TT	0.240	0.069	3.471	0.001	0.104	0.376	H5
	TS	0.615	0.057	10.739	0.000	0.502	0.728	H6
	TPHSQ	0.172	0.061	2.828	0.005	0.052	0.291	H1
	R^2^	0.942	*F* =1630.4444, *p* <0.0001					

*DV, dependent variables; IVs, independent variables; and S.E., standard error*.

This study verified the relationship between tourists’ trust in public health services and their satisfaction. The results showed that a significant positive effect exists between tourists’ trust in public health services and their satisfaction (*B*=0.677, *p*<0.05). The results supported H4.

Hypotheses 5 and 6 indicated that tourist trust and satisfaction significantly influence loyalty. The results showed that a significant positive correlation exists between tourist trust and loyalty (*B*=0.240, *p*<0.05) and a significant positive correlation is found between tourist experience satisfaction and loyalty (*B*=0.615, *p*<0.05). These results support H5 and H6.

### Testing Mediating Effect

To test the effect of multiple mediators on loyalty, the study conducted a series of mediator analyses using Hayes’ Model 6 and Bootstrap methods, and Table 6 shows the results for all paths (Hayes, 2012).

**Table 6:** Regression coefficients of serial mediation models estimated using PROCESS.

Model	Effect	S.E.	95% CI	
			Boot lower	Boot upper
TPHSQ→TT→TL	0.233	0.106	0.055	0.477
TPHSQ→TS→TL	0.404	0.087	0.224	0.561
TPHSQ→TT→TS→TL	0.210	0.055	0.116	0.337

*S.E., standard error*.

The results showed that the indirect effect of TPHSQ on loyalty was significant under the influence of chain mediators [*B*=0.847, 95% CI: (0.735, 0.966)]. The indirect effect of TPHSQ on loyalty was verified through the significant mediation of tourist trust [*B*=0.233, 95% CI: (0.055, 0.477)] and satisfaction [*B*=0.404, 95% CI: (0.224, 0.561)]. Thus, this result indicates that tourist trust and satisfaction play a partially mediating role in the effect of TPHSQ on their loyalty, thereby verifying H7 and H8.

The model in this study is a multi-mediator, which involves the relationship between two mediating variables, and these multiple mediating variables showed sequential characteristics and formed a chain of mediators. Therefore, the chain of mediators of tourist trust and satisfaction [*B*=0.210, 95% CI: (0.116, 0.337)] needs to be further explored and verified to have a significant effect.

## Discussion

## Impact of Tphsq on Tourist Loyalty

First, a significant positive impact is observed from the relationship between TPHSQ and loyalty and the results, which is consistent with the research results of (Jeaheng et al., 2020; Loke, 2020). In other words, if tourists perceive high-quality tourism public health services, then they are more likely to recommend to others or revisit. Specifically, Table 3 shows that among the 11 indicators, the respondents were sensitive to “Good natural ecological environment.” This finding shows that these tourists are satisfied with the environment of their most recent travel. In addition, the tourists were least sensitive to “There is an infirmary specially set up for tourists.” This notion can be explained by the fact that the tourists participating in this empirical study had different travel purposes. For tourists, whose main purpose is entertainment, they paid more attention to the basic public health services of the destination, such as the cleanliness of the environment and public toilets. They did not pay special attention to professional medical services. Moreover, a prediction could be proposed. For tourists who are for medical treatment or health, they will pay additional attention to professional medical and health services.

Then, this study not only verified the mechanism of the effect of TPHSQ on loyalty but also concluded that TPHSQ consists of two dimensions, namely, overall environmental image and public health facilities and management. This finding is consistent with the established findings of (Sun, 2018). Specifically, when tourists go to a destination, the first thing they perceive must be the environment and cleanliness of the destination (Breiby and Slatten, 2015; Hanafiah et al., 2019). For example, for natural destinations, if the ecological environment in the destination is not well protected or the water in the lakes and rivers is muddy, then such destinations may be meaningless to tourists. In addition, public health facilities and management are another important component of the quality of public health services in tourism, which is consistent with Raposo et al. (2009) in primary healthcare services. The number and layout of the public toilets in a destination, the maintenance of trash cans, and the way to report and complain about public health service problems are all factors that affect tourists’ experience (Milman, 2009).

### Mediating Role of Tourist Trust and Tourist Satisfaction

First, the TPHSQ affects tourist trust because public health services are different from other services in that they involve human health and safety, as evidenced by Maslow’s hierarchy of needs theory. In this theory, safety needs are the most sensitive needs of the human, in addition to physiological needs. Therefore, a satisfactory tourism public health service will allow tourists to conduct other experiences without fear (Loke, 2020). Second, the TPHSQ affects satisfaction, and this conclusion is not difficult to draw (Jeaheng et al., 2020). The reason is that tourists are involved in the production of tourism services, and they will use the perceived results of the process as a basis for their basic evaluation of service quality. Therefore, managers must design and provide services that meet expectations and satisfy needs to ensure the perceived effectiveness and satisfaction of tourists.

Furthermore, the current study confirmed a significant positive effect of tourist trust on satisfaction, which is consistent with the findings of Wu et al. (2016) and Amoako et al. (2019). Interestingly, the relationship between tourist trust and satisfaction has been in academic controversy, with some scholars arguing that satisfaction affects tourist trust (Song et al., 2014; Al-Ansi and Han, 2019; Martinez-Navalon et al., 2020). However, in this context, tourist trust affects satisfaction, which can be explained by the fact that tourism public health services are closer to the basic needs of tourists, such as health and safety. Although finding tourism public health services with high quality is not the main purpose for tourists, if they lose trust during travel, then satisfaction is even less likely to be achieved. In short, when tourists perceive that tourism public health services meet their expectations, they believe that the destination is trustworthy and reliable because their health and safety will be guaranteed during the trip. Then, they will be able to participate in other services with confidence, and their satisfaction will increase. Moreover, they will be more willing to recommend it to others or revisit it after the trip.

## Conclusion and Implications

### Conclusion

Tourism public health services are closely related to the health and experience of every tourist. In the post-epidemic era, if tourism companies can ensure that every tourist can receive high-quality public health services during travel, and then this can help regain travelers’ confidence and increase their loyalty and the recovery of tourism industry. This study aims to define and measure the TPHSQ and explore its impact on loyalty. The results showed that the TPHSQ consists of two dimensions, namely, overall environmental image and public health facilities and management. And it is positively correlated with tourist loyalty, and trust and satisfaction play an intermediary role in the TPHSQ–loyalty relationship. In addition, comprehensively, a single intermediary path based on satisfaction is better than a multi-chain intermediary path based on trust–satisfaction. The empirical results supported the theoretical model and hypothesis.

### Implications

Some scholars predicted that the panic caused by the COVID-19 outbreak may have a long-term impact on the tourism industry (Ugur and Akbiyik, 2020). Tourists are gradually lacking the confidence to travel, which is detrimental to the recovery of the tourism industry. Therefore, DMOs must adopt effective strategies to boost travelers’ confidence and help businesses recover on time after this public health crisis. One of the most effective strategies is to provide high-quality tourism public health services (Shi and Lu, 2020). Tourism destinations must focus on improving the TPHSQ and providing effective public health services, which in turn will improve tourist experience satisfaction, tourist trust, and loyalty. Therefore, we offer the following implications.

First, tourist destinations must change their attitude toward the overall environment and cleanliness. The cleanliness of the tourist environment is not the core of every travel and tourists are often attracted by other more beautiful landscapes or impressive services. However, as long as the tourists perceive the environment and sanitation below expectations, satisfaction will be greatly affected, especially during a public health crisis (Jeon and Kim, 2012; Wang et al., 2020). Therefore, tourist destinations should improve other service factors on the premise of ensuring a clean and healthy environment, such as ensuring good air and water quality and no garbage in the scenic area.

Second, tourist destinations must standardize public health facilities and management. Taking public toilets in scenic spots in China as an example, despite the implementation of the Toilet Revolution policy, unreasonable numbers, low convenience, and poor facilities in the public toilets still exist. In addition, even some of the toilets show a “different” situation, where the exterior decoration is neat and tidy, into the toilet but very poor hygiene (Sun et al., 2019). These cases have seriously affected tourists’ satisfaction, and likely tourists will not revisit or recommend the destination to other potential tourists. In addition, the epidemic has made tourists more concerned about their health and safety (Wen and Jiang, 2020). DMOs must establish a complete public health service system and learn to combine advanced technology or equipment to implement more effective public health service measures. Moreover, DMOs should help tourists obtain public health services safely and conveniently (Sun, 2018). Examples include the following: using robots for services during epidemic prevention and control to avoid direct contact with tourists; using the Internet to accurately calculate the use of public toilets to achieve its efficient use; or actively building a tourism public health information service system through brochures and guides maps, mobile APP, and other forms to provide tourists with convenient public health information services, such as the geographic location, distribution layout, and flow of public health service facilities.

## Limitations and Future Research

This study provides valuable management insights for the sustainable development of tourist destinations in the post-epidemic era. However, several limitations exist. One of them is that the current study collected data from Chinese tourists, so the research results may not be applicable to tourist destinations in other countries. Thus, future research can explore cross-cultural aspects for international or cross-cultural tourists. In addition, common problems related to the measurement errors of dependent and independent variables (IVs) exist in linear models. Finally, as the study validated a limited number of variables, future scholars can explore the relationship between the quality of tourism public health services and more other variables, such as tourists’ emotions and perceived risks.

## Data Availability Statement

The datasets presented in this article are not readily available but the raw data supporting the conclusions of this manuscript will be made available by the authors to any qualified researcher. Requests to access the datasets should be directed to Jiayu Han amber939999@163.com.

## Author Contributions

JH and YZ contributed to the conception of the study. JH collected and organized the data. JH and YZ contributed significantly to analysis and manuscript preparation. YZ and SC performed the data analyses and wrote the manuscript. RL and MZ helped perform the analysis with constructive discussions. MZ is responsible for the overall project. All authors contributed to the article and approved the submitted version.

## Conflict of Interest

The authors declare that the research was conducted in the absence of any commercial or financial relationships that could be construed as a potential conflict of interest.

## Publisher’s Note

All claims expressed in this article are solely those of the authors and do not necessarily represent those of their affiliated organizations, or those of the publisher, the editors and the reviewers. Any product that may be evaluated in this article, or claim that may be made by its manufacturer, is not guaranteed or endorsed by the publisher.

## References

[ref1] Al-Ansi A. Han H. (2019). Role of halal-friendly destination performances, value, satisfaction, and trust in generating destination image and loyalty. J. Destin. Mark. Manag. 13, 51–60. 10.1016/j.jdmm.2019.05.007

[ref2] Amoako G. K. Neequaye E. K. Kutu-Adu S. G. Caesar L. D. Ofori K. S. (2019). Relationship marketing and customer satisfaction in the Ghanaian hospitality industry an empirical examination of trust and commitment. J. Hosp. Tour. Insight. 2, 326–340. 10.1108/JHTI-07-2018-0039

[ref3] An M. Noh Y. (2009). Airline customer satisfaction and loyalty: impact of in-flight service quality. Serv. Bus. 3, 293–307. 10.1007/s11628-009-0068-4

[ref4] Anderson R. Karunamoorthy S. (2003). E-satisfaction and e-loyalty: a contingency framework. Psychol. Mark. 20, 123–138. 10.1002/mar.10063

[ref5] Aslam W. Arif I. Farhat K. Khursheed M. (2018). The role of customer trust, service quality and value dimensions in determining satisfaction and loyalty: an empirical study of Mobile telecommunication industry in Pakistan. Market-Trziste 30, 177–194. 10.22598/mt/2018.30.2.177

[ref6] Aydin S. Özer G. (2005). The analysis of antecedents of customer loyalty in the Turkish mobile telecommunication market. Eur. J. Mark. 39, 910–925. 10.1108/03090560510601833

[ref7] Bai K. Guo S. W. (2010). Study on the impact of inbound tourists’ emotional experience on their loyalty—a case study of Islamic traditional community in Huifang, Xi’an. Tourism Tribune 25, 71–78.

[ref8] Bitner M. J. (1990). Evaluating service encounters: the effects of physical surroundings and employee responses. J. Mark. 54, 69–82. 10.1177/002224299005400206

[ref9] Bitner M. J. (1992). Servicescapes: the impact of physical surroundings on customers and employees. J. Mark. 56, 57–71. 10.1177/002224299205600205

[ref10] Breiby M. A. Slatten T. (2015). The effects of aesthetic experiential qualities on Tourists’ positive emotions and loyalty: a case of a nature-based context in Norway. J. Qual. Assur. Hosp. Tour. 16, 323–346. 10.1080/1528008X.2015.1016591

[ref11] Camara R. F. M. (2019). The trust as a determinant of commitment, intention to revisit and the tourist’s recommendation to a destination: the case of Cancun. Ciencia Ergo-Sum 26, 1–12. 10.30878/ces.v26n3a1

[ref12] Chang K. C. (2014). Examining the effect of tour guide performance, tourist trust, tourist satisfaction, and flow experience on tourists’ shopping behavior. Asia Pac. J. Tour. Res. 19, 219–247. 10.1080/10941665.2012.739189

[ref13] Christmann A. Van Aelst S. (2006). Robust estimation of Cronbach’s alpha. J. Multivar. Anal. 97, 1660–1674. 10.1016/j.jmva.2005.05.012

[ref14] Cong L. C. (2021). Perceived risk and destination knowledge in the satisfaction-loyalty intention relationship: An empirical study of European tourists in Vietnam. J. Outdoor Recreat. Tour. 33:100343. 10.1016/j.jort.2020.100343

[ref15] Cronin J. J. Taylor S. A. (1992). Measuring service quality: a reexamination and extension. J. Mark. 56, 55–68. 10.1177/002224299205600304

[ref16] Deutsch M. (1958). Trust and suspicion. J. Confl. Resolut. 2, 265–279. 10.1177/002200275800200401

[ref17] Fauzi A. Suryani T. (2019). Measuring the effects of service quality by using CARTER model towards customer satisfaction, trust and loyalty in Indonesian Islamic banking. J. Islam. Mark. 10, 269–289. 10.1108/JIMA-04-2017-0048

[ref18] Fornell C. Larcker D. F. (1981). Evaluating structural equation models with unobservable variables and measurement error. J. Mark. Res. 18, 39–50. 10.1177/002224378101800104

[ref19] Guo A. X. Huang F. C. Li W. (2013). An empirical study on the most key drivers of revisiting intentions—comparison of perceived value, perceived attraction, tourist satisfaction and tourist trust. J. Jiangxi Univ. Financ. Econ, 38–40.

[ref20] Hair J. F. Black W. C. Balin B. j. Anderson R. E. (2010). Multivariate Data Analysis. Englewood Cliffs, NJ: Prentice Hall.

[ref21] Han H. Hyun S. S. (2015). Customer retention in the medical tourism industry: impact of quality, satisfaction, trust, and price reasonableness. Tour. Manag. 46, 20–29. 10.1016/j.tourman.2014.06.003

[ref22] Hanafiah M. H. Jasmi A. F. Razali A. H. M. Sulaiman M. S. (2019). The structural relationships of experience quality, tourist satisfaction and destination loyalty: the case of Pangkor Island, Malaysia. J. Nusantara Stud. 4, 186–210. 10.24200/jonus.vol4iss1pp186-210

[ref23] Hayes J. R. (2012). Modeling and remodeling writing. Writ. Commun. 29, 369–388. 10.1177/0741088312451260

[ref24] Imperial Hotel Osaka (2020). Information on Safety Measures for the Prevention of Novel Coronavirus Infections. Available at: https://www.imperialhotel.co.jp/e/osaka/news/info_coronavirus.html

[ref25] Iranmanesh M. Moghavvemi S. Zailani S. Hyun S. S. (2018). The role of trust and religious commitment in Islamic medical tourism. Asia Pac. J. Tour. Res. 23, 245–259. 10.1080/10941665.2017.1421240

[ref26] Isa S. M. Lim G. S. S. Chin P. N. (2019). Patients' intent to revisit with trust as the mediating role: lessons from Penang Malaysia. Int. J. Pharm. Healthc. Mark. 13, 140–159. 10.1108/IJPHM-10-2017-0056

[ref27] Jeaheng Y. Al-Ansi A. Han H. (2020). Impacts of halal-friendly services, facilities, and food and beverages on Muslim travelers’ perceptions of service quality attributes, perceived price, satisfaction, trust, and loyalty. J. Hosp. Mark. Manag. 29, 787–811. 10.1080/19368623.2020.1715317

[ref28] Jeon S. Kim M.-S. (2012). The effect of the servicescape on customers’ behavioral intentions in an international airport service environment. Serv. Bus. 6, 279–295. 10.1007/s11628-012-0136-z

[ref29] Lee S. P. Moghavvemi A. (2015). The dimension of service quality and its impact on customer satisfaction, trust, and loyalty: a case of Malaysian banks. Asian J. Bus. Account. 8, 91–121.

[ref30] Lee C.-K. Yoon Y.-S. Lee S.-K. (2007). Investigating the relationships among perceived value, satisfaction, and recommendations: the case of the Korean DMZ. Tour. Manag. 28, 204–214. 10.1016/j.tourman.2005.12.017

[ref31] Loke Z. (2020). Investigation of medical- and wellness tourists of a Hungarian spa to explore relationships between service quality, customer satisfaction and loyalty. Deturope Cent. Europ. J. Reg. Develop. Tour. 12, 102–118.

[ref32] Martinez-Navalon J. G. Gelashvili V. Saura J. R. (2020). The impact of environmental social media publications on user satisfaction with and trust in tourism businesses. Int. J. Environ. Res. Public Health 17:5417. 10.3390/ijerph17155417, PMID: 32731381 PMC7432117

[ref33] Maskey R. Fei J. Nguyen H.-O. (2018). Use of exploratory factor analysis in maritime research. Asian J. Shipp. Logist. 34, 91–111. 10.1016/j.ajsl.2018.06.006

[ref34] Melian-Alzola L. Martin-Santana J. D. (2020). Service quality in blood donation: satisfaction, trust and loyalty. Serv. Bus. 14, 101–129. 10.1007/s11628-019-00411-7

[ref35] Milman A. (2009). Evaluating the guest experience at theme parks: an empirical investigation of key attributes. Int. J. Tour. Res. 11, 373–387. 10.1002/jtr.710

[ref36] Oliver R. L. (2014). Satisfaction: A Behavioral Perspective on the Consumer. 2nd *Edn*. New York: Routledge.

[ref37] Paparoidamis N. G. Chumpitaz Caceres R. (2007). Service quality, relationship satisfaction, trust, commitment and business-to-business loyalty. Eur. J. Mark. 41, 836–867. 10.1108/03090560710752429

[ref38] Parasuraman A. P. Zeithaml V. Berry L. (1988). SERVQUAL: a multiple- item scale for measuring consumer perceptions of service quality. J. Retail. 64, 12–40.

[ref39] Peric G. Dramicanin S. Gasic M. (2020). Impact of service quality on satisfaction and loyalty of tourists in rural tourism of Sumadija and Western Serbia. Ekonomika Poljoprivreda-Economics of Agri. 67, 1071–1086. 10.5937/ekoPolj2004071P

[ref40] Pizam A. (1978). Tourism’s impacts: the social costs to the destination community as perceived by its residents. J. Travel Res. 16, 8–12. 10.1177/004728757801600402

[ref41] Plentz K. (1978). Protective vaccination in long-distance tourism as a problem of public health service (author's transl). Offentl. Gesundheitswes. 40, 497–506. PMID: 150580

[ref42] Priporas C. V. Stylos N. Vedanthachari L. N. Santiwatana P. (2017). Service quality, satisfaction, and customer loyalty in Airbnb accommodation in Thailand. Int. J. Tour. Res. 19, 693–704. 10.1002/jtr.2141

[ref43] Raposo M. L. Alves H. M. Duarte P. A. (2009). Dimensions of service quality and satisfaction in healthcare: a patient’s satisfaction index. Serv. Bus. 3, 85–100. 10.1007/s11628-008-0055-1

[ref44] Shi P. H. Lu M. M. (2020). Guaranteeing tourism health in the context of regular epidemic prevention and control—new perspectives and new innovations in addressing the impact of COVID-19 on tourism. J. Xinjiang Normal Univ. 41, 55–67. 10.14100/j.cnki.65-1039/g4.20200723.002

[ref45] Song H. J. Lee C. K. Kim M. Bendle L. J. Shin C. Y. (2014). Investigating relationships among festival quality, satisfaction, trust, and support: the case of an oriental medicine festival. J. Travel Tour. Mark. 31, 211–228. 10.1080/10548408.2014.873313

[ref46] Sun K. (2018). Research on the Evaluation of Rural Tourism Public Health Service Quality Based on Tourist Perception—A Case Study of Yuanjia Village. (Master’s degree), Northwest University, Available from Cnki.

[ref47] Sun R. Zhou R. Yuan Y. (2019). User experience design in scenic toilet for “toilet revolution.” Packag. Eng. 40, 232–238. 10.19554/j.cnki.1001-3563.2019.10.040

[ref48] Tanford S. Jung S. (2017). Festival attributes and perceptions: a meta-analysis of relationships with satisfaction and loyalty. Tour. Manag. 61, 209–220. 10.1016/j.tourman.2017.02.005

[ref49] The NY State Park. (2020). The NY State Park Guidelines You Need to Know During COVID-19. Available at: https://hvmag.com/coronavirus-covid-19-hudson-valley/ny-state-park-guidelines-rules-closed/

[ref50] Ugur N. G. Akbiyik A. (2020). Impacts of COVID-19 on global tourism industry: a cross-regional comparison. Tour. Manag. Perspect. 36:100744. 10.1016/j.tmp.2020.100744, PMID: 32923356 PMC7474895

[ref51] UNWTO. (2021). 2020 Global Tourism Revenue Loss Of 1.3 Trillion US Dollars. Available at: https://www.leddisplayls.com/news/2020-global-tourism-revenue-loss-of-1-3-trilli-42328960.html

[ref52] Wang C. Zhang J. H. Sun J. K. Chen M. Yang J. H. (2020). Public environmental facilities: hygiene factors for tourists' environmental behaviour. Environ. Sci. Pol. 106, 40–47. 10.1016/j.envsci.2020.01.009

[ref53] Warshaw P. R. Davis F. D. (1985). Disentangling behavioral intention and behavioral expectation. J. Exp. Soc. Psychol. 21, 213–228. 10.1016/0022-1031(85)90017-4

[ref54] Wen J. Jiang Y. (2020). Effects of COVID-19 on hotel marketing and management: a perspective article. Int. J. Contemp. Hosp. Manag. 32, 2563–2573. 10.1108/IJCHM-03-2020-0237

[ref55] Wu H.-C. Cheng C.-C. Ai C.-H. (2018). A study of experiential quality, experiential value, trust, corporate reputation, experiential satisfaction and behavioral intentions for cruise tourists: the case of Hong Kong. Tour. Manag. 66, 200–220. 10.1016/j.tourman.2017.12.011

[ref56] Wu H. C. Li T. Li M. Y. (2016). A study of behavioral intentions, patient satisfaction, perceived value, patient trust and experiential quality for medical tourists. J. Qual. Assur. Hosp. Tour. 17, 114–150. 10.1080/1528008X.2015.1042621

[ref57] Yoon Y. Uysal M. (2005). An examination of the effects of motivation and satisfaction on destination loyalty: a structural model. Tour. Manag. 26, 45–56. 10.1016/j.tourman.2003.08.016

[ref58] Žabkar V. Brenčič M. M. Dmitrović T. (2010). Modelling perceived quality, visitor satisfaction and behavioural intentions at the destination level. Tour. Manag. 31, 537–546. 10.1016/j.tourman.2009.06.005

[ref59] Zhou Y. H. Maumbe K. Deng J. Y. Selin S. W. (2015). Resource-based destination competitiveness evaluation using a hybrid analytic hierarchy process (AHP): the case study of West Virginia. Tour. Manag. Perspect. 15, 72–80. 10.1016/j.tmp.2015.03.007

