# Supplementary material for: Service Quality in Tourism Public Health: Trust, Satisfaction, and Loyalty
Source: Front Psychol. 2021 Aug 30;12:731279. doi: 10.3389/fpsyg.2021.731279 (PMC8435890; doi:10.3389/fpsyg.2021.731279)
Supplement: Supplementary file 1 [file Table_1.DOCX]

**Appendix A. Measurement items for study**

| Measurement items |  |
| --- | --- |
| Tourism public health service quality |  |
| TPHSQ1 | Good natural ecological environment |
| TPHSQ2 | There are no disorderly piles, dislocations, or constructions |
| TPHSQ3 | The place to visit is clean and tidy |
| TPHSQ4 | Buildings and various equipment are in good condition |
| TPHSQ5 | Public toilets have clear instructions and high convenience |
| TPHSQ6 | Public toilets have complete facilities and services and are very clean |
| TPHSQ7 | The number and layout of public toilets are very reasonable |
| TPHSQ8 | There is an infirmary specially set up for tourists |
| TPHSQ9 | There are various ways of reporting and complaining about health issues, which are convenient and efficient |
| TPHSQ10 | Environmental supervision departments and personnel are very responsible |
| TPHSQ11 | Can easily obtain effective public health information services |
| Tourist trust |  |
| TT1 | I trust completely this tourism destination. |
| TT2 | This tourism destination is mainly concerned with the tourists’ interests related to their health and safety. |
| TT3 | The public health services provided by the tourism destination are reliable. |
| TT4 | I feel that I can rely on the staff in this tourism destination to serve good public health service. |
| TT5 | I believe that I can trust the public health' service ability of the staff. |
| Tourist satisfaction |  |
| TS1 | Overall, I am satisfied with this travel. |
| TS2 | Compared with the expectations, I am satisfied with this travel. |
| TS3 | Compared with other similar experiences, I am satisfied with this travel. |
| Tourist loyalty |  |
| TL1 | I would like to visit this destination again. |
| TL2 | If I have the opportunity, I will recommend this destination to friends/relatives. |
| TL3 | If I have the opportunity, I will promote the positive information of the destination to others. |
